# Research on Electrocatalytic Materials for Hydrogen Evolution and Oxygen Evolution

**DOI:** 10.3390/ma18184232

**Published:** 2025-09-09

**Authors:** Jiangtian Li, Deryn Chu

**Affiliations:** U.S. Army DEVCOM Army Research Laboratory, Adelphi, MD 20783, USA

There is growing interest in pursuing more sustainable energy sources to replace traditional fossil fuels. Hydrogen stands out as a promising option because it can store and release energy efficiently and produces only water when used as fuel [1]. Conventional hydrogen production primarily relies on natural gas reformation, a process that demands substantial investment in infrastructure and transportation. As an alternative, water electrolysis using electricity offers a more adaptable alternative. This method splits water into hydrogen and oxygen, producing high-purity hydrogen suitable for applications such as fuel cells, where additional purification is unnecessary [2,3]. Furthermore, when driven by indigenous energy sources such as solar, wind, or hydropower, water electrolysis can strengthen energy resilience and provide capabilities that reduce—and potentially eliminate—reliance on fossil fuel supply. However, the inherent kinetic barriers to hydrogen evolution reactions (HERs) and oxygen evolution reactions (OERs) demand highly efficient and cost-effective electrocatalysts to make them viable at scale. To overcome this, researchers are working to develop advanced materials that can speed up these reactions efficiently and affordably, but this remains a major challenge in the field [1,2,3,4]. This Special Issue, ‘Research on Electrocatalytic Materials for Hydrogen Evolution and Oxygen Evolution’, gathers ten original research papers that highlight recent advances in catalyst design, synthesis, and evaluation for the HERs and OERs used in water splitting. The contributions span a range of strategies, from atomic-level surface modification and heterostructure engineering to waste valorization and electrolyte design, offering insights into our fundamental understanding of this topic, as well as practical implementations.

For large-scale applications, the cost of catalysts must be considered alongside their catalytic activity. Currently, state-of-the-art water-splitting catalysts still heavily rely on noble metals. Therefore, the development of efficient, non-precious metal catalysts is in high demand. In this Special Issue, Leiva-Guajardo et al. demonstrate the use of industrial copper slag waste as a HER electrode in saline water electrolysis. Its porous structure facilitates ion transport and enhances HER kinetics while offering environmental remediation through waste reuse. Bojîncă et al. compared Ni catalysts produced via pulsed current deposition from Watts and citrate baths. Ni deposited from citrate exhibits finer grains and superior HER performance, highlighting electrolyte control as a key synthesis lever. In cases where noble metals are used, developing strategies to minimize their loading is essential for reducing costs. Single-atom catalysts (SACs) have emerged as a promising solution, offering advantages such as uniform active sites, high atom utilization, and excellent catalytic activity. Wu et al. designed a sequential underpotential deposition approach to anchor Cu and Pt single atoms onto N-doped graphene, achieving high turnover frequencies in alkaline HERs due to maximized active site exposure. Zhang et al. construct Mo_2_C nanoparticles encapsulated in MoS_2_ layers to form synergistic interfaces, enhancing charge transport and hydrogen adsorption, resulting in robust acidic HER performance. To investigate the underlying mechanism, Li, Chu et al. employed XPS depth profiling, revealing that P-doping facilitates the reconstruction of sulfide catalysts into Ni-O species during alkaline HERs. This transformation boosts catalytic activity by modulating the electronic structure and *d*-band center. In addition to catalyst improvement, Perović et al. introduced Ni, Zn, and Mo ions into an alkaline electrolyte as ionic activators, resulting in enhanced HER activity. This in situ activation method reduces overpotentials and energy costs, showcasing a potential low-cost improvement route.

Regarding the alkaline oxygen evolution half-reaction, transition-metal-based layered double-hydroxides (LDH) represent one of the most active classes of OER catalysts. In this Special Issue, Li et al. investigate the effect of Fe, Mn, Cu, and Zn doping on NiCo-layered double-hydroxides. Fe-doped catalysts exhibit the best OER performance due to their improved conductivity and nanosheet dispersion. This study offers significant contributions to the development of efficient electrocatalysts for OERs, advancing our understanding of key design principles for enhanced catalytic performance. Yang et al. fabricated composite catalysts using a short plasma process, forming a defect-rich interface between NiFe-LDH and PbO_2_ on reduced graphene oxide. This structure exhibits excellent OER activity with low overpotentials. Although an OER is thermodynamically more favorable in alkaline electrolytes, it remains a significant scientific challenge in acidic environments, especially when paired with a Pt cathode for acidic water splitting, due to issues related primarily to catalyst stability. Kuang et al. presented a CeO_2_-Ir hybrid catalyst with strong interfacial coupling on carbon nanotubes. This structure achieves high OER activity and durability in acidic media, using significantly reduced Ir content. Lastly, Sun et al. developed porous NiTiO_3_-BiOBr composites that exhibit efficient photocatalytic activity under visible light. A type-II heterojunction facilitates charge separation and extends photocatalytic lifetime.

The ten contributions showcased in this Special Issue illustrate the breadth and depth of current research efforts in the field of electrocatalyst development, from atomic-level design to large-scale waste reuse. They offer valuable insights into the structure–activity relationships, reaction mechanisms, and performance engineering concerns present in diverse electrochemical conditions. We thank all of the contributing authors, peer-reviewers, and the *Materials* editorial team for their dedication and support. We hope that this Special Issue will serve as a vital reference for researchers working to realize clean and efficient hydrogen technologies.
